# Particulate Bone Allograft Incorporation in Regeneration of Osseous Defects; Importance of Particle Sizes

**DOI:** 10.2174/1874325000701010019

**Published:** 2007-12-18

**Authors:** Theodore I Malinin, Ellen M Carpenter, H. Thomas Temple

**Affiliations:** 1 Tissue Bank, Department of Orthopaedics, University of Miami, Miller School of Medicine Miami, Florida, USA; 2 Mannheimer Foundation, Homestead, Florida, USA; 3 Department of Psychiatry, University of California Los Angeles, School of Medicine, Los Angeles California, USA

## Abstract

Packing of bone defect with particulate allografts is a commonly performed clinical procedure. However, the ideal size of bone particles used to fill bone defects is ill-defined. For this reason the study of biology of bone allografts with different particle sizes has been performed. Standard size bone defects in the femur and the tibia of experimental animals were filled with freeze-dried cortical bone allografts with particle sizes of 1-2mm, 800-500μm, 500-300μm, 300-90μm, 250-125μm, 125-106μm, 106 to 75μm and 75-25μm. Unfilled defects and those filled with autologous bone served as controls. Cortical bone was chosen because it produced better clinical results than did cancellous bone. Likewise freeze-dried particulate bone effected more rapid healing than did frozen bone. Numerical scores were assigned to each defect based on the gross, radiographic and histomorphometric studies. Particles in the range of 300 to 90 microns produced rapid healing by direct ossification. Particles below 100µm had a significantly reduced osteogenic potential. Particles in the range of 75-25μm failed to heal the defects all together. Healing of defects packed with particles larger than 300μm was slower than with 300 – 90 μm grafts. Rapid healing of bone defects packed with particulate bone allografts in the range of 300 to 90μm indicates such allografts can be used effectively in the filling of bone defects. This is of clinical relevance.

## INTRODUCTION

Filling of unicameral and aneurysmal bone cysts with bone allograft was studied by Spence *et al*. who compared cancellous and cortical bone particles and demonstrated superiority of cortical versus cancellous bone in healing of the cysts [1, 2]. In these studies freeze-dried bone was used. In experimental studies, when freeze-dried bone was compared to its frozen counterpart it produced more rapid and uniform healing [3]. Freeze-dried particulate bone was also used in obliterating defects following excision of benign and low grade malignant tumors [4,5]. Spence employed bone particles 2-4 mm in size. Other investigators have used bone graft particles of various sizes with no unanimity of opinion regarding optimal particle sizes. Some authors of experimental studies state that smaller particles induce more bone formation than do the larger ones [6]. Others hold an opposite view [7-10]. From the practical standpoint it would be advantageous to use extremely small powder like particulate bone graft since it is easily compacted and thus would obliterate the entire cavity. However, it has not been shown whether or not true bone powder is effective in promoting rapid healing of bone defects. Regardless of whether an allograft is cortical or cancellous it is generally agreed that the size of the graft correlates with the graft incorporation, bone healing and biomechanical properties of the graft [11,12]. However, it is not known which particle sizes induce the highest rates of healing. Therefore we have performed a comparative animal study in which we filled standard osseous defects with bone particles of different sizes and determined the effect of the same on osteoinduction, osteoconduction and rapidity of the healing of bone defects. We hypothesized there may be a relationship between particle size of the allograft and its ability to promote bone healing. For this study we employed freeze-dried cortical allografts because the same were shown clinically effective, and because freeze-dried particulate bone allografts healed faster than did their frozen counterparts [2,3]. In planning this investigation we selected a non-human primate model, and used a well described and validated combined cortico-cancellous defect technique [2,13,14]. Non-human primates, including baboons, have been used extensively in musculoskeletal transplantation studies because of their anatomical similarities to humans [15-18].

## MATERIALS AND METHODS

The study design included packing of standard intraosseous defects with bone allografts composed of different particle sizes and quantitating healing of the defects by radiographic, gross, and histomorphometric measurements. Thirty-eight healthy, young and mature outbred male and female baboons (Papio hamadryas) with an average weight of 8.35 kg SD=0.69 were used in the experiments. Skeletal maturity was determined by the closure of the epiphyseal plate in the distal femurs. The animals were kept in colonies in the open–air enclosures. Only one defect per animal was filled with a specific allograft being tested. In total there were 68 defects. Fifty nine were in animals sacrificed at 6 weeks and represented in Table **2**. These were 6 unfilled controls, 6 filled with autograft, 6 filled with 1-2mm cubes, 7 filled with 500-800µm particles, 4 filled with 500-300µm particles 9 filled with 300-90µm particles, 5 filled with 250-125 µm particles, 6 filled with 125-106 µm particles, 4 with 106-75µm particles, and 6 with 75 to 25µm.

To determine an appropriate sample size for the study power analysis was performed using Lenth Java applets computer software for power and sample size [19]. A score of 45, assigned under the scoring system used and derived from comparing control preparations to 1-2mm samples was considered significant, as it indicated some initial bone healing. This value reflects a readily identifiable difference between control preparations that show no healing (assigned score of 0) and the onset of osteogenesis induced by clinically accepted 1-2mm cortical bone. Therefore an effect size of 45 as the standard for significance was set. Assuming a difference in values of the mean = 45 with a standard for significance deviation of 10, to reach statistical significance of p<0.05, a sample of n=4 would give 99.5% power. Using the same assumptions, a minimal sample of 2 would yield a power of 64.6%. Since the intent of the study was to identify bone particles that lead to optimal bone healing, these conditions would reveal effects relevant to outcome.

The animals were fed a standard Purina Monkey Chow (PMI Nutrition International, LLC, Brentwood, MO) diet supplemented with fruit. Before surgical procedures were initiated, the research protocols were approved by Institutional Animal Care and Use Committee. All surgical procedures were carried out in an operation room. Throughout the experiments, the animals were under the care of veterinarians in accordance with USDA regulations and NIH recommendations.

Aseptically excised bone from lower extremities of six animals not included in the group of 38 experimental animals was used for the preparation of allografts. Bone was cleaned of soft tissues, washed with agitation with warm saline to remove bone marrow and fat, wrapped in cotton towels, and sealed in plastic bags. Washing of the graft with removal of bone marrow and extraosseous fat allowed for compacting of the graft material in the defect [11]. Bone was then rapidly frozen in liquid nitrogen vapor. Aliquots of liquid nitrogen frozen bone were placed into a freeze-dryer chamber using aseptic precautions. The freeze-dryer chambers were sterilized with ethylene oxide and then aerated. Bone was freeze-dried for 5 days with a freeze dryer condenser at-50° to -60°C and shelves at -30° to -20°C. Before removing freeze-dried bone from the apparatus, the shelves were heated to 25°C. The freeze-drying regimen used produced a product with gravimetrically determined residual moisture of 3% to 5%. Freeze-dried bone was ground incrementally in an industrial (Mill- Tek, Tekmar Dohrman, Cincinnati, OH) grinder without overheating. The bone was then sieved through USA Standard Testing sieves. Preparations with particle sizes were produced as follows: 800-500µm, 500-300µm, 300-90µm, 250 to125µm, 125 to 106µm, 106 to 75µm, and 75 to 25µm. These were compared with “crushed bone 1 to 2mm in size. Eighty to 85% of particles in these preparations were within the specified range. The 15% to 20% were smaller.

Animals immobilized with ketamine (5 mg/kg of body weight) were intubated and anesthetized with isoflurane. Vital signs were monitored throughout aseptically performed surgical procedures. Preoperatively, animals were given 25 mg/kg cefazolin. Cefazolin was also administered twice a day for 3 days postsurgery. After the operation, animals were maintained individually in cages for 3 to 4 days. Analgesia was provided with burprenorphine (0.01 mg/kg of body weight) every 12 hours for 1 to 2 days. Animals were then returned to their respective colonies. Food and water was given ad libitum.

Distal femurs and proximal tibias were approached through anterior incisions. Corticomedullary defects measuring 9 to 10mm in diameter and 15mm in depth were created with intermittent burring with saline irrigation in the metaphysis- diaphysis of the distal femur and the proximal tibia. Sometimes the defects involved the epiphyseal line. The defects extended into medullary canal. Consequently, there was considerable bleeding. Hemorrhage was controlled by packing the cavity with bone graft which acted as an excellent hemostatic agent. Control (unpacked) defects were packed with gauze sponge which was left in place under pressure until the bleeding subsided. One or two defects were placed in the distal femur and the proximal tibia in each animal. In animals with small tibias no defects were made in the same tibia because of fear of fracture. For the present study fifty nine defects were made. Only one defect per animal was filled with a particular type of allograft under study. Autologous bone was bone slurry obtained from burring of defects. Since only one specific type of allograft was implanted per animal each defect was considered an independent sample. The technique of creating and filing the defects with preparations under study consisted of burring holes with frequent irrigation with saline, measuring the depth of the defect, filling it with particulate allograft and tampering the same. Unfilled defects served as negative controls and those filled with autografts obtained with burr from two or more sites were positive controls. Roentgenograms (anteroposterior and lateral) were obtained postoperatively and at 6 weeks. Twenty nine of 38 animals were sacrificed at 6 weeks and 9 at 12 weeks. Euthanasia was achieved by an intravenous injection of FATAL-PLUS (Vortech Pharmaceutical, Dearborn, Michigan). Before sacrifice at 6 weeks limbs of experimental animals were radiographed. Defects with smaller particles (75-25µ) were not healed at this time. Therefore a decision was made to maintain representative animals for total of 12 weeks to determine if these would heal by that time. These included 2 animals each with 75-25 µm and 300-90 µm particles, one animal each with 1-2mm, 800-500 µm and 500-300 µm particles and 2 controls. Femurs and tibias were dissected and frozen in dry ice. The bones were then sectioned with a diamond saw, photographed, and radiographed. Bone specimens were fixed in 10% formalin- Earle’s balanced salt solution mixture, decalcified in Perenyi’s fluid (10% nitric acid and 0.15% chromic acid in 30% alcohol), embedded in paraffin, and sectioned at 5 to 7µm. Sections were stained with hematoxylin and eosin, PAS-hematoxylin, Romanowski-Giemsa, and Masson’s trichrome stains. The results were expressed as a combination of gross, radiographic and histomorphometric data. Results were averaged for each experimental group. A total score of 100 was assigned to bone which was normal grossly, radiographically, and microscopically. A score of 0 would be assigned to an unaltered defect if the cavity was completely unhealed and remained open (Table **1**).

Histomorphometric measurements were based on color conversion generated by translating grey scale image to 20 color representations to visualize distinct differences which correlated with histologically apparent new bone formation using NIH image 1.62. Area within a defect occupied by newly formed bone was measured using Image ProPlus

Version 5.0 for Windows. The grading was performed by two authors(TM and HTT) on coded specimens. It is of course, obvious that specimens with large particles could not be mistaken for those with smaller particles. However, specimens with particles 506-300µm, 300-90µm-250-125µm and 125-106µm had similar appearance. Interobserver variability was within 15-20%. In arriving at a final score the differences were averaged. Data from the scores for experimental and control groups, as previously defined, were compared with each other using a one-tailed Student’s t-test assuming two-sample unequal variance. Value of p<0.05 was considered significant.

## RESULTS

**Fig. (2):**
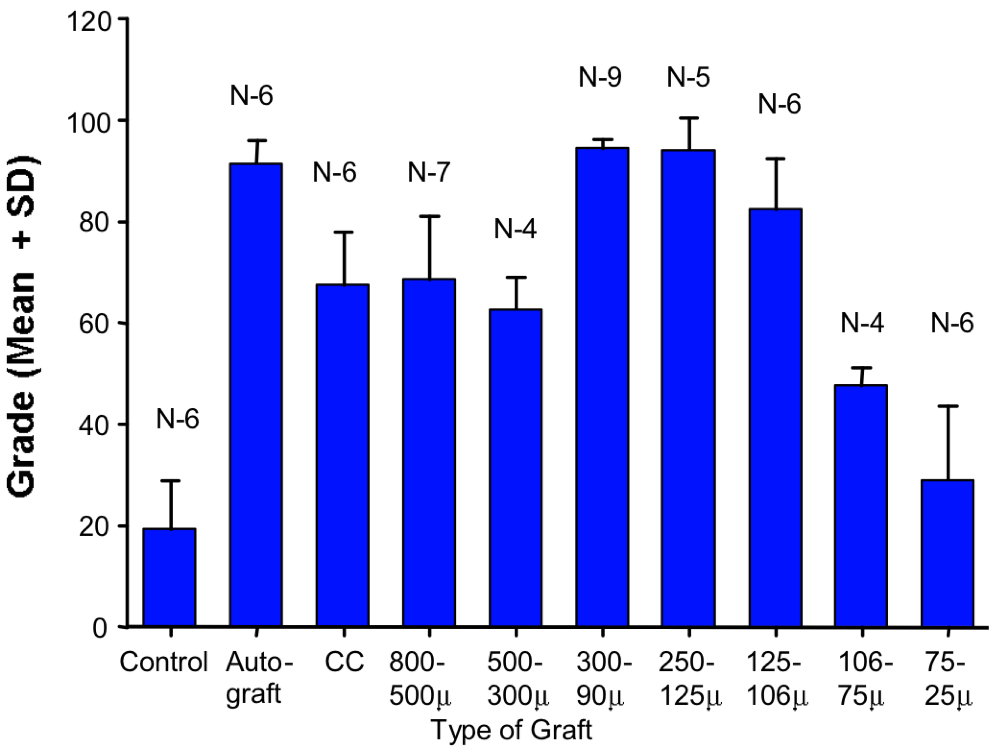
Graphic representation with error bars of the assessment of defect healing using a scale of 100. Statistical significance of differences between experimental groups was established using Student’s t-test. Statistical data is summarized in Table **2.**

The results of the study demonstrate, at six weeks, clear differences in the ability of allograft bone particles of different sizes to induce new bone formation in bone defects packed with these (Fig. **2**).

At twelve weeks defects filled with particles 75-25 µm remained unhealed. Control (unfilled) defects were healed less than one fourth. Similar observations were made on sheep [14]. The methods employed in the study allowed for determination of quantitative difference in the replacement of the defined bone defects with regenerating bone. The experimental animal model used was constant. With the exception of one wound dehiscence there were no wound or other complications. There were statistically significant differences between the healing of control (unfilled) defects and defects filled with bone particles of all sizes, other than those filled with the smallest particles of 75 to 25µm. On the other hand only particle sizes of 300-90, 250-125, and 125-106µm were comparable to autografts. All other allografts tested showed scores statistically significantly lower than those for autografts.

The negative control animal (empty defect) demonstrated very little bone repair at 6 and 12 weeks. In the periphery of the defect, there was some new bone formation. The major portion of the defect was filled with gelatinous material. Bone defects filled with 1 to 2mm and 800 to 500-µm particles were similar. Allograft particles were recognized grossly, radiographically, and microscopically. The healing process with new bone formation proceeded slowly. In the center, the particles were surrounded with fibrous connective tissue. In the periphery, some particles showed Howship’s lacunae with osteoclasts and rims of osteoblasts in between these. Defects filled with 500 to 300µm particles were only partially healed with amorphous material in the center. Bone particles inadvertently spilled into muscles were resorbed. Defects filled with microparticulate graft (300-90µm) were replaced with newly formed cancellous bone. Individual particles were surrounded and replaced by directly ossified newly formed bone with intense osteoblastic activity. Many of the particles were permeated by vascular channels lined with osteoblasts. Defects filled with particles between 250 and 125µm and those filled with particles 125 to 106 µm resembled those filled with 300-90µm particles, except that the particles themselves were not prominent. Defects packed with grafts composed of smaller particles, 106 to 75µm and 75 to 25µm appeared strikingly different. These were filled with amorphous acellular material with virtually no new bone formation except in the periphery of the defects At twelve weeks all defects other than those filled with 106-75µm and 75-25µm particles, and the control defects have healed to a significant degree. However, a small number of samples in this group did not allow for separate quantification of the data.

## DISCUSSION

The study was performed to determine whether or not there were differences in the biology of freeze-dried bone allografts of different particle sizes. Contrary to expectations bone sizes 106-75µm and those 75 to 25µm were ineffective in promoting the healing of bone defects in which they were packed. On the other hand bone allografts with particle sizes in the range of 300-90µm were comparable to autograft. Therefore, our hypothesis that regeneration of the intraosseous defects packed with particulate bone allograft is related to the particle size of the graft particles appears to be correct. The results published in the literature are not uniform. Marx *et al. *reported that undecalcified cancellous bone particles, comparable in size to the ‘crushed bone’ used in our study did not perform as well as did autologous grafts, but they did heal eventually without producing an inflammatory response [10]. Observations made in the present study are similar. Glowacki *et al. *found that deminerialized bone powder with particle size of <75µm induced bone formation in cranial defects of rats to a larger extent than did larger particles [6]. Gepstein *et al.* in experiments also performed in rats, on the other hand, state that longer demineralized particles (up to 450µm) were effective in bridging defects in the radius [20]. The difference can be explained by the difference between membranous and endochondral bones. Baboon model has been used extensively in studying biology of bone grafts [16-18, 21, 22]. Thus our choice of the animal model was appropriate. However, the limitation of this and similar studies is the cost. The limitations of the study also include reliance on morphologic assessment of the bone healing in a single point of time, but it must be borne in mind that nearly all that is known about osteogenesis has come from histologic observation of transplanted bone examined at a single point in time [23]. It must be noted that specimens, other than those filled with 106-75µm and 75-25µm particles, examined at 12 weeks post transplantation showed significant healing of the defects, indicating a difference in the speed of repair. Unfortunately the number of animals in this time framework was too small to allow for quantification.

The present experiments were performed in a manner that allowed for comparative assessment of bone graft incorporation. The model was similar to that originally described by Maatz, and then used by other investigators [3, 13, 14]. Its main advantage is that it allows for quantification of the bone replacement in a circular defect of known delineations. The primate models for the study of bone regeneration offer an advantage because in many ways, these animals approximate human skeletal structure. Ripamonti described a baboon model for the study of calvarial regeneration [18]. Kohless *et al. *evaluated several allograft preparations in filling of periodontal defects in the same animals [21]. Baboons were also used for study of massive distal femur allograft transplantation [22] and for the transplantation of smaller osteoarticular allografts [16]. Boden *et al. *investigated spinal fusions in adult rhesus macaques [15]. The comparatively large size of baboon bones allowed for the placement of defects without fracturing either femurs or tibias. Random placement of the defects ensured avoidance of transplanting allografts being tested into preferred sites. In the present model, bone allografts with particles of sizes enumerated in Materials and Methods showed differences in the healing patterns and osteogenesis. Our results show that non-demineralized bone particles, below 106µm induce little osteogenesis and may, in fact, inhibit bone healing. This suggests various bone powder preparations may be subject to the same limitations. The mechanism may be analogous to the inhibition of osteoclastic activity by small particles of hydroxyapatite [23]. Another possible explanation might be that densely packed bone powder forms a solid, cement-like mass which prevents vascular ingrowth. Bone allografts in the 300-90µm range induced simultaneous activity of osteoclasts and osteoblasts, were rapidly vascularized, and surrounded and replaced by newly formed bone. In other words, active particles underwent direct ossification. Osteoconduction by these grafts can be attributed to compact particle placement, which allows for revascularization, leaching out of BMP and other growth factors from the bone particles and by retention of intraosseous lipids necessary for transport of BMP [24-26]. Osteogenesis induced by particulate allografts in nonhuman primate model suggests these can be effective in filling skeletal defects in humans. To distinguish biologically effective particles from the bone powders the term microparticular allografts might be useful.

## Figures and Tables

**Fig. (1):**
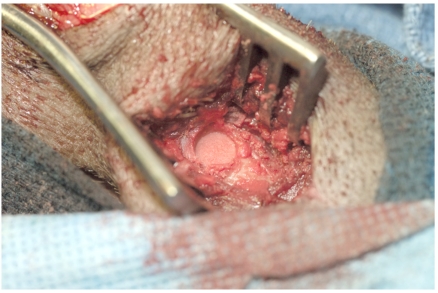
A cylindrical defect filled with particulate bone preparation. The defect was created by intermittent burring with irrigation. The defect was packed with particulate bone allograft which produced hemostasis.

**Fig. (3):**
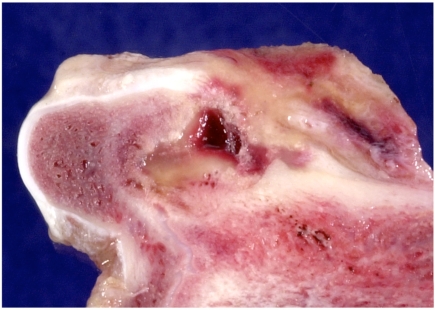
Control preparation 6 weeks after creating a defect; the defect remains unhealed with only a thin rim of new bone in the periphery.

**Fig. (4):**
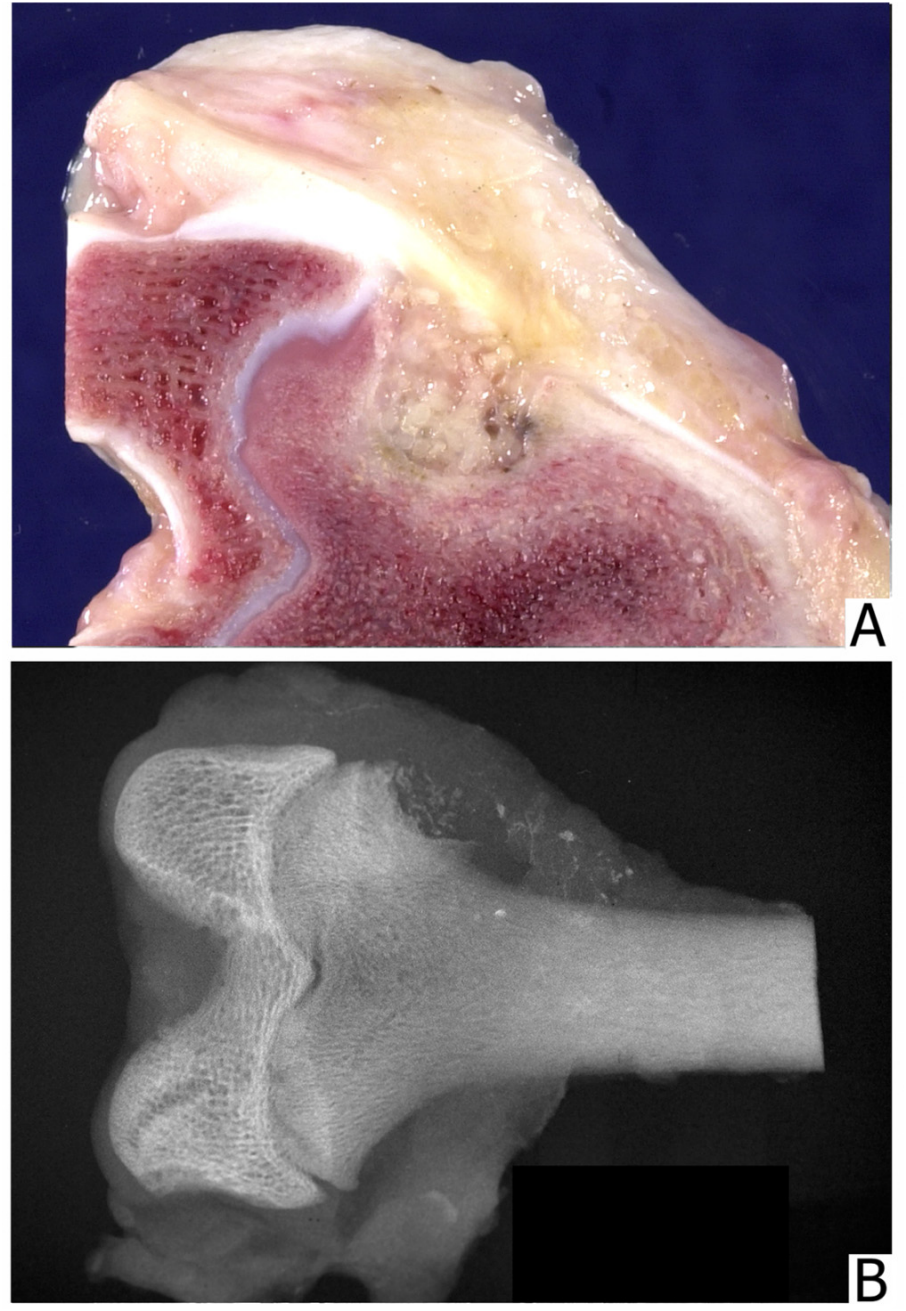
**(A)** Defect filled with 1-2 mm bone particles; particles are recognizable grossly. **(B)** A radiograph of a defect filled with 800-500 µm particles. The edges of the defect are distinct and the granules are recognizable.

**Fig. (5):**
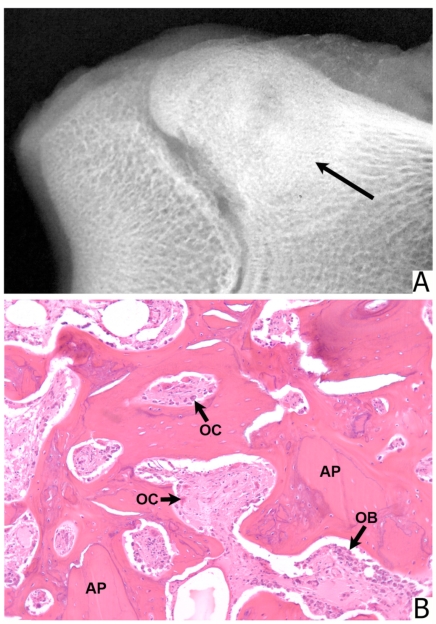
Defect filled with 300 to 90 µm microparticulate bone allograft. **(A)** Radiograph shows new bone formation which reaches the epiphyseal line. (arrow) (**B**) Histologic section from the center of the defect. Allograft particles (AP) are revascularized and are undergoing direct ossification. Both osteoclasts (OC) and osteoblasts (OB) are present. (Stain, hematoxylin and eosin; original magnification, x 100).

**Fig. (6):**
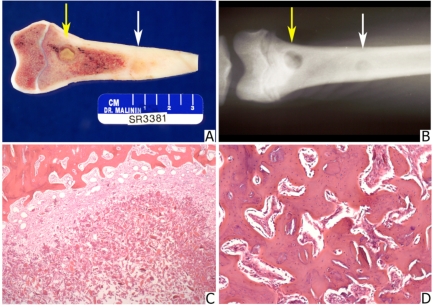
**(A)** A photograph showing distal femur with two defects. The most distal defect (arrow) was filled with 75 to 53 µm allograft particles. The defect is largely filled with yellowish amorphous material. The proximal defect (arrow) has been filled with 250 to 125 µm particles. It has healed. **(B)** A radiograph of the same specimen shows an unhealed distal defect (arrow) and healed proximal defect (arrow). **(C)** A histologic section of the distal defect. The defect is filled with acellular material and granulation tissue in the periphery (Statin, hematoxylin and eosin; original magnification, x35). **(D)** A histologic section from the center of the proximal defect. Newly formed bone is similar to that shown in Fig. **(5)** (Stain, hematoxylin and eosin; original magnification, x 100).

**Table 1.** Method of Grading Healing of Bone Defects

Gross	Points Awarded
Defect open; unhealed Defect unhealed, filled with fibrous tissue or unaltered graft material Defect filled with new bone; less than 25% Defect filled with new bone to 50% Defect filled with new bone 50 to 75% Defect filled with new bone 75% or more Defect completely filled; indistinguishable from surrounding bone	0 0 5 10 15 20 25
** Radiographic** Defined clearly visible; unfilled Defined clearly visible; filled with clearly recognizable graft particles Defect partially filled; less than 25% Defect filled to 50% Defect filled over 50% Defect filled with new bone, with trabecular bridging and unrecognizable host-graft interface Defect indistinguishable from surrounding tissue	0 0 5 10 15 20 25
** Histomorphometry** No new bone present Unaltered graft material occupied over 50% of defect Graft particles surrounded by thin rims of new bone Particles replaced by new lamellar bone with many particles still visible Over one half particles replaced with new lamellar bone; revascularization evident Dense bone trabeculae present with moderate osteogenesis; some endochondral bone formation or direct ossification Cavity filled with newly formed bone trabeculae lined with osteoblasts; bone marrow formation	0 10 20 20 30 40 50
**Maximum score**	**100**

**Table 2.** Summary of the Statistical Analysis Comparing Individual Experimental Groups (*=P<0.05. NS = not significant)

	Auto	CC	800–500	500-300	300-90	250-125	125-106	106-75	75-25
Control	*	*	*	*	*	*	*	*	NS
Autograft		*	*	*	NS	NS	NS	*	*
CC			NS	NS	*	*	NS	*	*
800-500				NS	*	*	NS	*	*
500-300						*	*	NS	NS
300-90						NS	NS	NS	*
250-125							NS	*	*
125-106								*	*
